# Endometrial stem cells in regenerative medicine

**DOI:** 10.1186/1754-1611-8-20

**Published:** 2014-08-01

**Authors:** Javad Verdi, Aaron Tan, Alireza Shoae-Hassani, Alexander M Seifalian

**Affiliations:** 1 Centre for Nanotechnology and Regenerative Medicine, UCL Division of Surgery & Interventional Science, University College London (UCL), London NW3 2QG, UK; 2 Applied Cell Sciences Department, School of Advanced Technologies in Medicine, Tehran University of Medical Sciences, Tehran, Iran; 3 UCL Medical School, University College London (UCL), London, UK; 4 Royal Free London NHS Foundation Trust Hospital, London, UK

**Keywords:** Endometrial stem cells, Menstrual blood stem cells, Endometrial regenerative cells, Endometrium, Regenerative medicine

## Abstract

First described in 2004, endometrial stem cells (EnSCs) are adult stem cells isolated from the endometrial tissue. EnSCs comprise of a population of epithelial stem cells, mesenchymal stem cells, and side population stem cells. When secreted in the menstrual blood, they are termed menstrual stem cells or endometrial regenerative cells. Mounting evidence suggests that EnSCs can be utilized in regenerative medicine. EnSCs can be used as immuno-modulatory agents to attenuate inflammation, are implicated in angiogenesis and vascularization during tissue regeneration, and can also be reprogrammed into induced pluripotent stem cells. Furthermore, EnSCs can be used in tissue engineering applications and there are several clinical trials currently in place to ascertain the therapeutic potential of EnSCs. This review highlights the progress made in EnSC research, describing their mesodermal, ectodermal, and endodermal potentials both *in vitro* and *in vivo*.

## Introduction

Cells in the earliest developmental stages in the embryo can generate embryonic and extra-embryonic tissues [1]. The ability to generate other cell types is known as potency. In the inner cell mass of the blastocyst in the embryo, the cells pluripotent, meaning they can give rise to the ectoderm, mesoderm, and endoderm lineages (entire organism). Pluripotent stem cells include embryonic stem cells (ESCs) from the inner cell mass of blastocysts, epiblast derived stem cells from embryos after implantation, embryonic germ cells (EGCs) from primordial germ cells, embryonic carcinoma cells (ECCs) derived from germ cells tumors, and germ line stem cells from testicular tissue [2-4]. As the reproductive cycle progresses, cell potency decreases and only a small fraction of cells retain their potency, namely adult stem and progenitor cells. These are limited to tissue generation within specific lineages. Adult stem cells (ASCs) are found in numerous human tissues including: intestines [5], muscles [6], skin [7], blood [8], nervous system [9-11], endometrium [12], heart, liver [13,14], dental pulp, adipose tissue, synovial membrane, umbilical cord blood, amniotic fluid [15,16], and the endometrium [17]. In comparison to pluripotent stem cells, ASCs are considered safer for therapeutic purposes and several are currently used in clinical trials. The concept of using ASCs over embryonic stem (ES) cells in regenerative medicine has recently gained traction. The rationale for this trend include: (1) The complicated control of the culture conditions of ESCs; (2) the existence of several intermediate stages before reaching terminal differentiation of ESCs; (3) Teratoma formation is a major obstacle for clinical development of ESCs; (4) immunological rejection of cells derived from ES cells to the recipient and (5) ethical scrutiny involved in ESCs application.

### Endometrium and endometrial stem cells (EnSCs)

**Figure 1:**
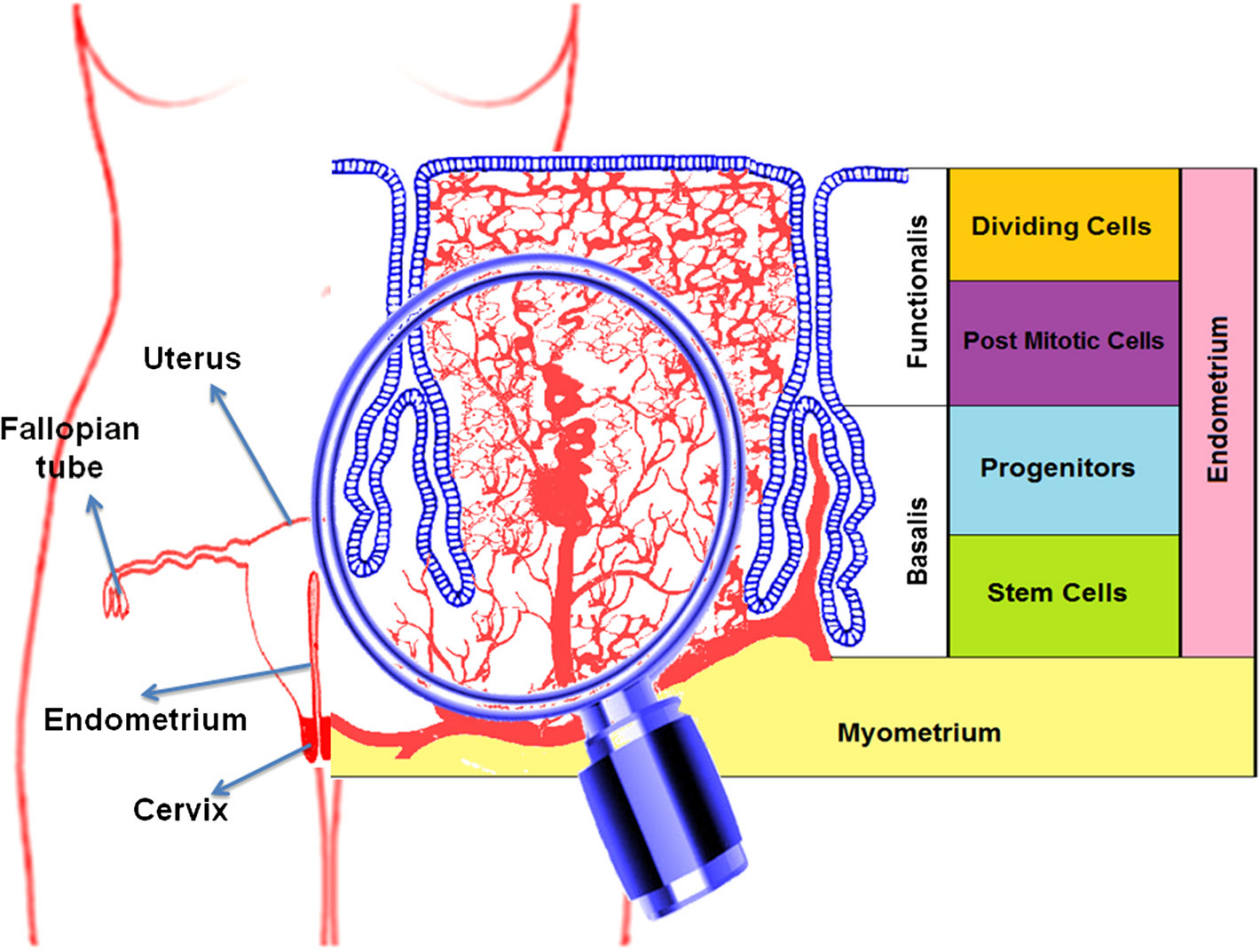
**The endometrium.** Location of endometrial stem cells in human endometrium. In human endometrium, epithelial progenitor cells are located in the base of the glands in the basalis, while endometrial stem cells are located near blood vessels even in functionalis.

The human endometrium is an extraordinary model of controlled tissue remodeling, unparalleled in other organs, which grows about 7 mm within one week in every menstrual cycle [18]. At this rate, there is a very rapid rate of angiogenesis for approximately 500 cycles within a tightly controlled manner in a woman’s lifetime. The human endometrium, derived from the mucosal lining of the fused paramesonephric tubes during embryogenesis, is a dynamic tissue (Figure 1). It is comprised of two major zones: (1) the functionalis, a transient layer containing glands extending from the surface epithelium as well as the supportive stroma, and (2) the basalis, comprised of the basal region of the glands, stroma, supporting vasculature, and lymphoid aggregates. Progenitor cells are located in the basal layer (Figure 1) of human endometrium [19]. These rapidly proliferating cells (transient cells) then move to the functional layer [20] and actively participate in the regeneration and remodeling of the endometrium. Endometrial mesenchymal stem cells (MSCs) have been identified in the endometrium (lining of the uterus). Indeed, cloning of human endometrial cells confirmed the existence of endometrial epithelial progenitor cells for the first time in 2004 [21]. There are three distinct endometrial stem cells including: epithelial progenitor cells, MSCs and endothelial progenitor cells [22]. Recent studies reported the isolation of multipotent EnSCs from menstrual blood [23,24] or endometrial biopsies [25]. These cells are called endometrial regenerative cells (ERCs), and are heterogenic and have morphological differences among their different isolation sites. Stromal cells have been cultured from menstrual blood [24,26], suggesting that endometrial MSCs are shed during menstruation [22] but epithelial cells have not been detected [27], suggesting that epithelial progenitors may not be shed during menstruation and more likely reside in the basalis. During the proliferative menstrual phase, the endometrium prepares for recruiting new EnSCs to cover the new blood vessels. The variation in the numbers of endothelial progenitor cells and stem cell derived factor-1 is a pivotal factor for release and homing of stem cells [28]. These EnSCs have properties similar to bone marrow or adipose tissue stem cells. They proliferate and differentiate throughout the estrous cycle and during pregnancy under the control of the ovarian hormones. Clonogenic MSCs are responsible for regenerating the endometrium, and they exist in peri-menopausal women, postmenopausal women, and women on oral contraceptives [29]. Irregular function of these SCs may contribute to the pathogenesis of endometriosis and the growth of tissue outside the uterine cavity, causing dysmenorrhoea, subfertility and endometrial carcinoma [30]. Stem cells that are ectopically distributed through lymphovascular metastasis [31] may also contribute to the pathogenetic process, because their high proliferation promotes rapid clonal expansion [32].

The progenitor cells in the endometrium has a high proliferative potential that can generate 6 × 10^11^ cells from a single cell, with the ability to differentiate into large cytokeratin-expressing structures when cultured in Matrigel [17], comprising laminin, collagen IV and heparan sulfate proteoglycan. It was used instead of mouse embryonic fibroblast feeder layers for ES cells proliferation. Our data shows that mesenchymal stem cells from endometrium can be grown extensively both *in vivo* and *in vitro*. These cells are not tumorigenic in nude mice and could be passaged approximately 40 times *in vitro*.

**Figure 2:**
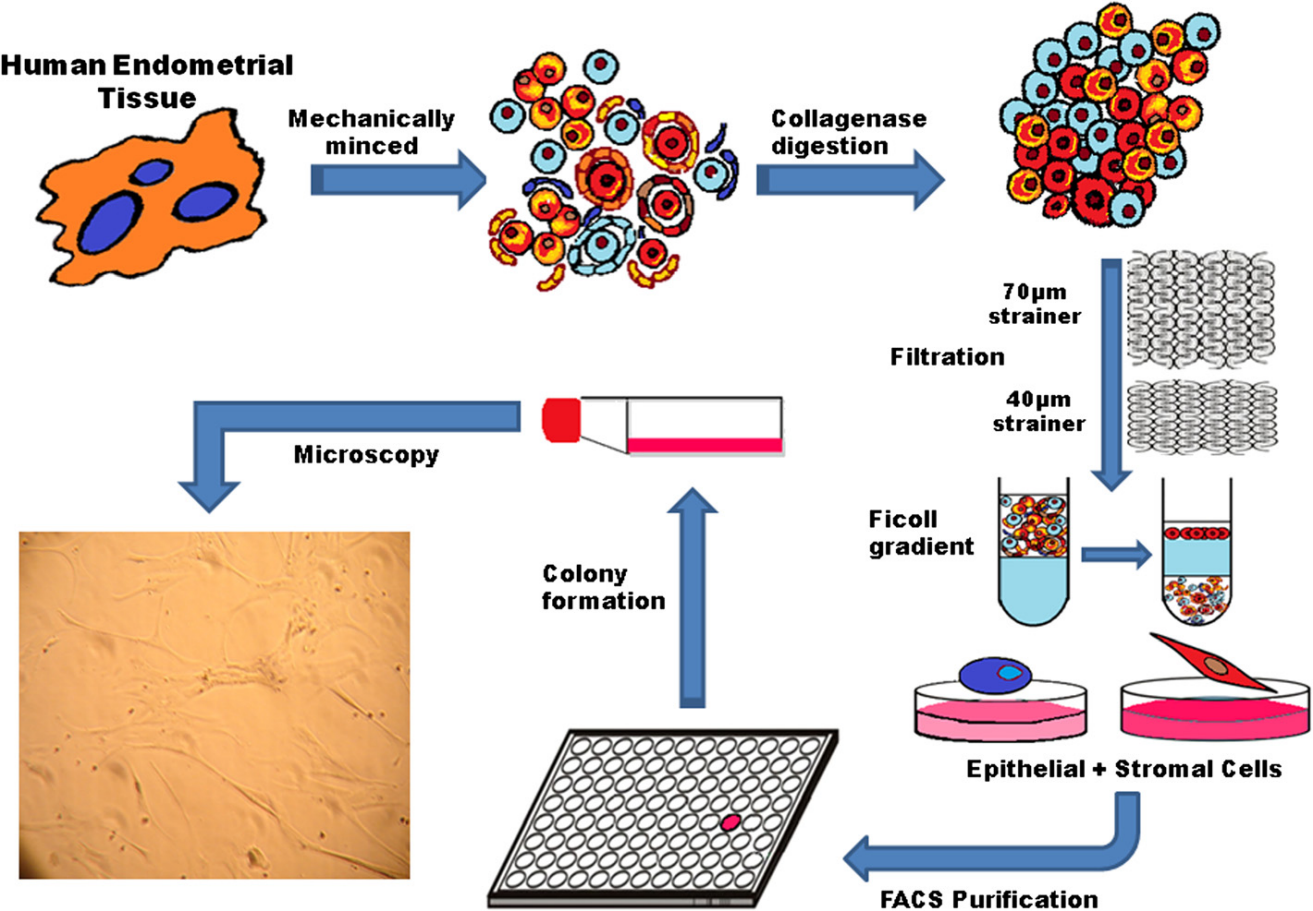
**The endometrial stem cell isolation.** A diagram that shows the stepwise isolation of endometrial stem cells by collagenase digestion, then serial filtration through 70 μm and 40 μm meshes, ficoll separation and selection the colony forming cells.

The fact that about 600,000 hysterectomies are performed yearly in the United States creates a potential source of endometrial cells [33]. Studies have indicated that EnSCs reside in the superficial layers accessible by endometrial biopsy [34,35]. Indeed, endometrial biopsies may represent a viable technique in harvesting EnSCs, as they are routinely performed for gynecologic purposes, and does not impair endometrial function. Thus, endometrial tissue specimens from women who are not receiving hormonal therapy are suitable for isolation of stem cells (Figure 2).

Endometrial stem cells may be derived from fetal stem cells or bone marrow stem cells, including haemopoietic SCs, MSCs and endothelial progenitor cells [36-38]. The human endometrium shows expression of the pluripotency factors Sox-2, Oct-4 and Nanog. Sox-2 co-localized with telomerase, contribute to the immortality of embryonic stem cells [39]. Reprogramming of differentiated cells to an induced pluripotent stem (iPS) cell is through induction or increasing in the expression of Oct-4, Sox-2, Klf-4, and c-Myc [40] that easily could be achieved from EnSCs that already express these factors. As a result, iPS cells from EnSCs have been established as early as 12 days after transduction rather than the usually reported 4 weeks for other cell types [41]. As patient- or disease-specific biomedical research using iPS cells becomes more widespread, the endometrium may play a key role as reprogrammable cells into donor- or disease-specific pluripotent cell lines for the female population.

### Markers of human EnSCs

**Figure 3:**
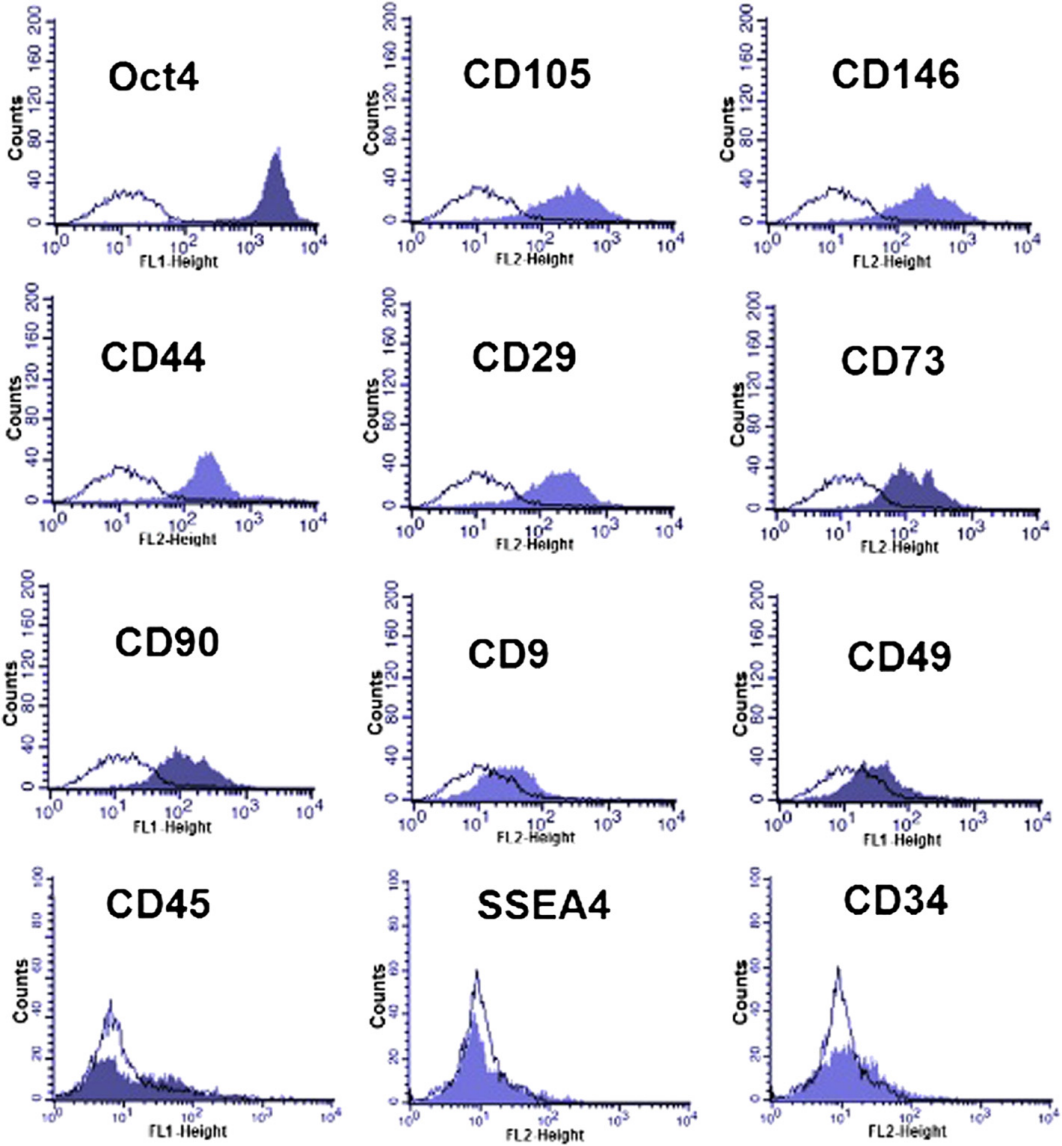
**Flow-cytometric analysis of endometrial stem cell markers.** Further phenotypic analysis of EnSCs after enrichment in culture. Expression of Oct4, CD90, CD105, CD146 and CD44 have more percentage of EnSC markers.

Flow cytometric analysis of important endometrial stem cell markers are indicated in Figure 3. Cultured EnSCs express typical markers used for bone marrow stem cells; CD9, CD13, CD14, CD29, CD31, CD44, CD73, CD90, CD105, CD117, CD133, CD146 [42], but not STRO-1, CD31 (endothelial) and CD34 (haemopoietic stem cell and endothelial) [43]. The EnSCs could be purified on the basis of their co-expression of two perivascular markers CD140b and CD146 [44]. Cells with a hematopoietic stem cell phenotype (CD34 + CD45+) co-expressing CD7 and CD56 have been identified in human endometrial cell suspensions and may be lymphoid progenitors [45]. Musashi-1, an RNA-binding protein in neural stem cells and an epithelial progenitor cell marker that regulates self-renewal signaling pathways, has been recognized in human endometrium. NAC1 is a transcription repressor that is involved in self-renewal and the maintenance of pluripotency in embryonic stem cells [46]. Ishikawa et al. demonstrated that NAC1 was over-expressed in the normal cyclic endometrium in the early and mid proliferative phases [47]. MSI1 and NOTCH1, which maintain SCs in an undifferentiated state, are expressed in EnSCs. Tissue non-specific alkaline phosphatase, also immune-localizes to a perivascular location in human endometrium and may be useful for the prospective isolation of EnSCs [48]. Leucine rich repeat containing G protein-coupled receptor-5 (Lgr-5) is also expressed in human endometrial epithelial progenitor cells and endometrial [49] stromal cells [50]. W5C5 is a single marker for purifying EnSC, which differentiates into adipogenic, osteogenic, chondrogenic, and myogenic cell lineages [51]. One-third of these EnSCs expressed α-smooth muscle actin (αSMA) but not CD31, suggesting that they are vascular smooth muscle cells and that they occupy a peri-vascular niche.

### Immunogenicity of EnSCs

Utilizing ES cells in regenerative therapy is associated with many hazards. Without adequate ES cells screening procedures, patients would face tissue rejection similar as rejection of organ transplants. A solution to this problem is to create patient specific stem cells. Unlike ES cells, adult stem cells are not rejected by the immune system [52]. The human endometrium has unique immunological requirements. In pregnancy, the endometrium must tolerate the invading embryo, which expresses both paternal and maternal antigens. In 2012, Peron et al. demonstrated that endometrial MSCs were able to suppress neuro-inflammation in a mouse model of multiple sclerosis. This suppression is dependent on the secretion of anti-inflammatory cytokines such as IL-10 and IL-27, and also the expression of indoleamine-2,3-dioxigenase [53]. Menstrual blood SCs were tested for allogeneic response in culture with peripheral blood mononuclear cells (PBMN). The SCs were analyzed at a 1:2 stimulator (mitomycin C treated PBMN) to responder cell (menstrual blood SCs) ratio for 6 days. The menstrual blood SCs demonstrated a moderately weak stimulatory response in a mixed lymphocyte reaction (MLR). An emerging bank of data allows for the classification of the mesenchymal stem cell from bone marrow as immunosuppressive cell source derived from studies with human cells [54,55]. Some studies support that SC administration is a potential way to suppress tumor growth. It has been demonstrated that human skin-derived progenitor cells have selective tropism for malignant tissues. Also more interestingly, these cells have the ability to inhibit tumor growth [56]. MSCs could selectively integrate into gliomas after intravascular or local delivery [57-59]. ERC administration inhibits C6 tumor growth and its administration associated with reduced neovascularization [60]. Despite the angiogenic potential of ERC in the hindlimb ischemia model, these data support a paradoxical tumor inhibitory activity of ERC. Further studies are needed to determine the qualitative differences between physiological angiogenesis, which seems to be supported by ERC and tumor angiogenesis which appeared to be inhibited. Other studies have demonstrated that MSCs directly secrete tumor inhibitory factors [61,62].

### Potency and differentiation of EnSCs

**Figure 4:**
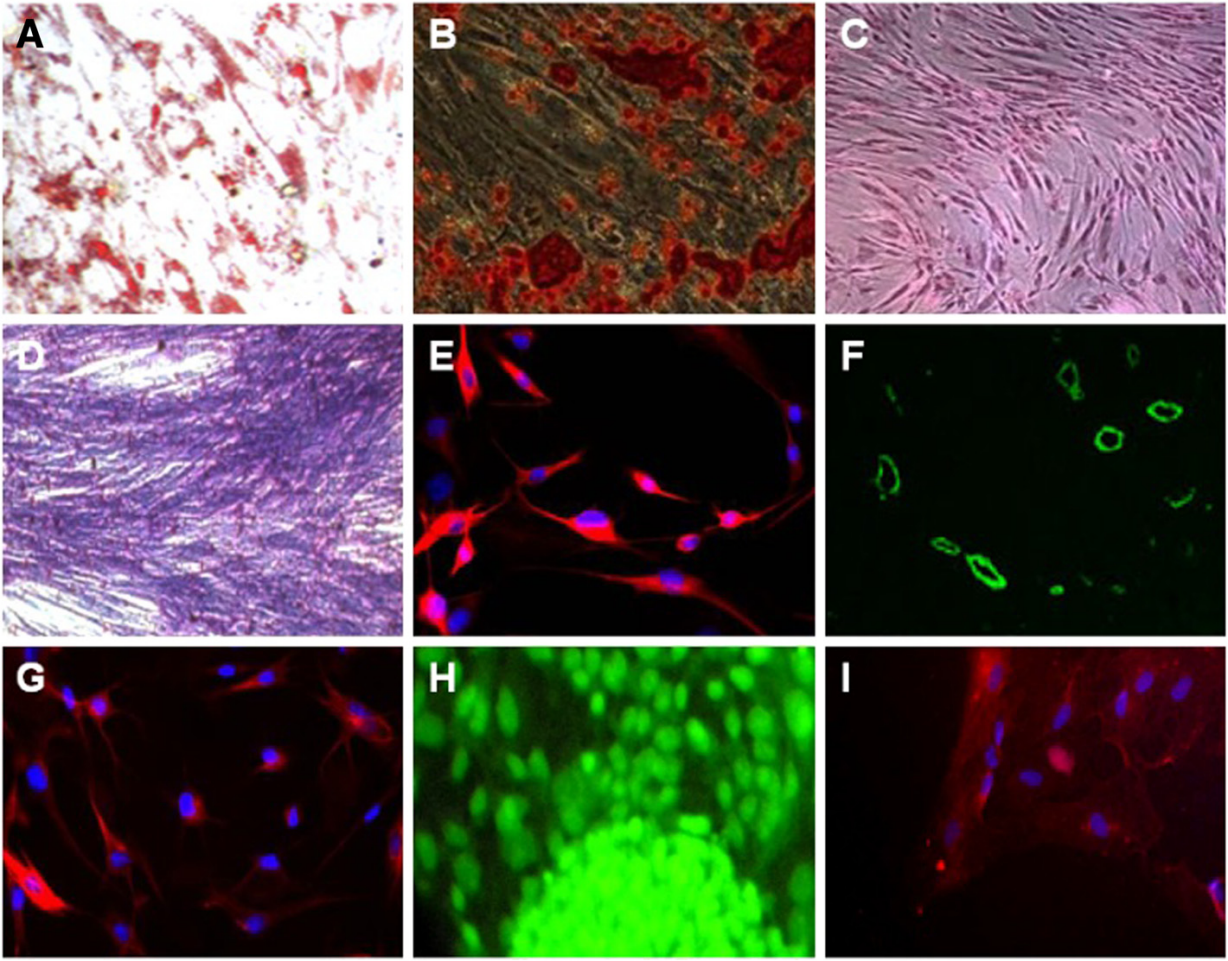
**Multipotent differentiation of endometrial stem cells.** Endometrial stem cells were cultured under appropriate differentiation media as described in box1-15 and assessed for differentiation using the indicated staining methods. **A)** Adipocytic differentiation, the red color indicates lipid vacuoles stained by Oil-Red. **B)** Osteocytic differentiation, red indicates calcium stained by Alizarin Red and **C)** Von-Kossa staining of osteoclasts differentiated from EnSCs. **D)** Chondrocytic differentiation, the blue color resulted from Alcian blue staining. **E)** Neuronal differentiation, the red conjugated mAb indicates pre-neural marker Nestin stain. **F)** Angiogenesis of EnSCs, the green indicated CD31 stain. **G)** Oligodendrocytic differentiation, the red conjugated mAb indicates receptor interacting protein (RIP). **H)** Hepatocytic differentiation, green indicates albumin stain. **I)** Myocytic differentiation, red indicates α-actinin stain.

The main features of mesenchymal stem cells are their ability for self-renewal, colony-forming ability, and differentiation into mesoderm-derived lineages including adipogenic, chondrogenic and osteogenic differentiation [63-65]. Endometrial SCs that are persistent in the uterine endometrium from the fetal to the postmenopausal period [66] were found to differentiate into adipocytes, osteoblasts and chondrocytes as well [67,68] (Figure 4). The modulation of p38 and c-jun might play an important role for the differentiation and proliferation of normal human endometrial cells [69]. These differentiations can identify by positive staining, with Oil Red O (for lipid droplets), alizarin red (for osteogenesis), von Kossa (for calcified extracellular matrix), and Alcian blue (for sulfated proteoglycans), respectively. Clonogenicity of EnSCs does not vary from the proliferative to secretory stage of the menstrual cycle, or between active, cycling and inactive endometrium for both epithelial and stromal cells [29].

### Identification and isolation of differentiated cells

Research pertaining to stem cell biology in the female reproductive tract is still in its infancy, and although surface markers of prospective isolation of human endometrial stromal colony-forming cells (putative endometrial stromal/progenitor cells) have recently identified [17,44], there remains a need for definitive markers of both myometrial and endometrial stem cells for more selective isolation and enrichment. Complete characterization of uterine stem/ progenitor cells will improve our understanding of the mechanisms supporting physiological regeneration of the female reproductive tract. In addition, such studies will enhance our understanding of uterine cancer, hyperplasia, endometriosis, leiomyomas and adenomyosis. Indeed, our data and the published observations from other laboratories have enabled us to propose a novel hypothetical model for eutopic and ectopic endometrial regeneration. Finally, availability of these stem cells suggests new approaches to reconstruction of the human uterus and perhaps other organs as well.

The final step for processing stem cells before transplantation would be the identification and isolation of the differentiated cells. Recognition and selection of desired cells can be done via reverse transcriptase polymerase chain reaction, flow cytometry or fluorescence-activated cell sorting (FACS). Unfortunately, these methods would destroy many of the differentiated cells, thereby rendering them unusable. Also, flow cytometry and FACS is dependent on the presence of specific markers on the cell surface, which are not always displayed on every type of differentiated cells [70]. Thus, a more viable technique for identification of differentiated cells can be made with the transient transfection of a reporter gene into a stem cell source. The reporter gene can encode a fluorescent (green fluorescent protein) or a drug selectable protein. The transient transfection would be favorable for human cell therapy because the reporter is only meant for selection purposes.

### Comparison between EnSCs, MSCs, and umbilical cord blood stem cells

Genetic profiling of EnSCs and bone marrow MSCs revealed that they had similar (although not identical) cytokine production, miRNAs, and gene expression [71]. It was reported that EnSCs express higher levels of ICAM-1 and IL-8, while bone marrow MSCs express higher levels of TGFβ1, TGFβ2, IL-6, and vascular endothelial growth factor (VEGF). This observation suggests that EnSCs may play a role in acute inflammation, and may be more suitable for tissue engineering applications. It was also reported that, compared to MSCs, EnSCs had a 27-fold higher level of PDGF-BB, and 14-fold higher level of angiopoietin. This indicates that EnSCs may activate alternative angiogenesis pathways. However, more studies are required to ascertain if EnSCs stimulate higher levels of angiogenesis than bone marrow MSCs *in vivo*. It was shown that EnSCs produced collagen type II and proteoglycans when cultured on nanofibrous polycaprolactone material, suggesting their cartilaginous tissue differentiation potential [72]. It was also observed that proliferation of EnSCs was faster than bone marrow MSCs, and EnSCs express the pluripotency marker OCT-4, but lack the MSC marker STRO1 [72]. Umbilical cord blood was first used in bone marrow transplant in a patient with Fanconi’s anaemia in 1988 [73]. Considering that a large proportion of haematopoietic stem cells are found in the fetal circulation, there is also a correspondingly large proportion of haematopoietic stem cells in the umbilical cord. Bone marrow MSCs are distinct from haematopoietic stem cells, as they are CD45^-^. Mature bone marrow MSCs are a heterogeneous population of cells that are able to support haematopoiesis, and capable of differentiating into different lineages such as neural cells [74,75], muscle [76-78], liver cells [79,80], among many others. Various studies have reported that a single mesenchymal progenitor cell is responsible for all lineages are produced from bone marrow MSC *in vitro*. It has been shown that bone marrow MSCs contain more mesenchymal progenitor cells when compared to umbilical cord blood stem cells. Indeed, the proportion of MSCs within umbilical cord blood is extremely low and difficult to detect [81]. Nevertheless, more studies comparing EnSCs, MSCs and umbilical cord blood stem cells are required to accurately ascertain their similarities and differences.

### EnSCs in regenerative therapeutics

To date, EnSCs have been used in several pre-clinical and small animal studies. The regenerative potential of EnSCs was first seen in an experimental model of Duchenne muscular dystrophy in immunodeficient mdx mice, whereby EnSCs that were transplanted into atrophied skeletal muscle fibres contributed to muscle repair [26]. Although the exact mechanism of repair has not been elucidated, it was postulated that cell fusion and *in situ* differentiation might have been involved, in part due to the observation that the transplanted EnSCs homed to peri-muscle fibre regions and promoted angiogenesis. The concept of angiogenesis was also supported by another study, where EnSCs were improved critical limb ischaemia induced by femoral artery ligation [82].

The use of EnSCs to treat myocardial infarction in a murine model was also seen. In this study, EGFP-labelled EnSCs were grafted into the infarct area of nude rat hearts, which subsequently differentiated into α-actinin^+^, troponin^+^ striated cardiac muscle cells [83]. Furthermore, it was observed that a significantly larger reduction in infarct area was seen in animals treated with EnSCs, compared to control bone marrow MSCs.

Gargett et al., the first group that reported the existence of EnSCs in 2004 [21], are currently developing an autologous tissue engineered scaffold using artificial meshes and EnSCs for the treatment of pelvic organ prolapse, and was tested *in vivo*, on an animal skin wound repair model [84,85]. Results indicated that EnSCs promoted neovascularization, increased tissue integration, reduced chronic inflammation, increased deposition of collagen fibres and distensibility of the mesh at 3 months post-implantation. They also found that EnSCs downregulated foreign body reactions and enhanced mesh integration, indicating they have a role to play in modulating tissue response with regards to implanted foreign materials.

Our group has also advocated the tissue engineering approach to the application of EnSCs. To this end, we have fabricated nanofibrous silk-collagen fibres that were seeded with EnSCs for the reconstruction of urinary bladder wall for women [86]. We had also shown that EnSCs were capable of differentiating into smooth muscle cells [86], potentially functioning as an autologous cell source for bladder tissue engineering.

Intraperitoneal delivery of EnSCs were also studied in a murine model of encephalomyelitis [53]. It was founds that EnSCs exerted a an anti-inflammatory effect, as evidenced by a lower number of infiltrating mononuclear cells in the lesions, upregulation of IL-10 and IL-27 in the spleen, and reduced recruitment of Th1 and Th17 cells in the central nervous system.

EnSCs were also used to demonstrate neural regenerative capabilities, whereby reduced neuronal cell death was seen when oxygen-deprived primary neuronal cell cultures were exposed to EnSCs [87]. It was also observed that EnSCs released neuroprotective trophic factors such as neurotrophin-3 (NT-3), brain-derived neurotrophic factor (BDNF), and VEGF. The *in vivo* part of the study was done in a murine model of ischaemic stroke, whereby injection of EnSCs resulted in significantly lower histological and behavioural impairments. It was reported that EnSCs exerted a trophic effect, releasing factors that promoted survival of neural cells.

The use of EnSCs to treat glioma was observed in a murine model. In this study, EnSCs were administered intravenously in a murine model of intracranial glioma. Results revealed a reduction of tumour size of almost 50%, possibly due to its anti-angiogenic effects [60].

The applications of EnSCs have also been reported in several human studies. The first reported use of EnSCs was demonstrated by Zhong et al. [88]. Clinical-grade menstrual blood-derived EnSCs have been used in a small Phase I clinical trial of 4 patients suffering from multiple sclerosis, whereby EnSCs were delivered via intravenous and intrathecal routes. Results showed no immunological reactions or adverse side effects after 1 year [88].

Another human study involved a patient suffering from Duchenne muscular dystrophy that was given intramuscular injections of EnSCs. Follow-up observations reported no adverse effects even after 3 years, and increased muscle strength and decreased respiratory infections was also reported [89].

The third reported use of EnSCs in human was a patient with congestive heart failure, who was given intravenous administration of EnSCs. Results revealved that ejection fraction of the patient increased from 30% to 40%, decreased basic natriuretic peptide values (Pro-BNP), and decreased Minnesota Living with Heart Failure Questionnaire score at 1-year follow up [90].

### The promise and limitations of EnSCs

EnSCs are an attractive source of stem cells for regenerative therapeutics as they are easily obtainable and easily expandable in culture, as has been demonstrated to be safe for clinical use. Protocols and methods for extraction and isolation of EnSCs are well established, as purified EnSCs can be obtained using magnetic bead sorting using the W5C5/SUSD2 marker. In addition, clinical-grade good manufacturing practice (cGMP) are currently being developed for culture expansion of EnSCs, and have been tested in animals. However, there is a lack of published information on the exact cGMP protocols in place for the production of EnSCs. This is compounded by the fact that there is no general scientific consensus regarding specific MSC markers to detect EnSCs; rather, researchers rely on the ability of MSCs to adhere to plastic. Hence, the purity of EnSCs is not guaranteed as the cultures could potentially contain fibroblasts.

EnSCs can be obtained from menstrual blood; hence no invasive procedures are needed to harvest these cells. A menstrual cup is used to collect menstrual blood over several hours on days 2 to 3 of the menstrual period. Although there is a potential risk of infection via vaginal contact, there have been no reports of any complications after antibiotic use.

Although the ability of EnSCs to re-integrate into tissue *in vivo* has been demonstrated, there is a theoretical risk that endometriosis could develop from using EnSCs. However, none of the animal model studies have reported this. Nevertheless, it is an aspect of EnSC application that warrants attention. Indeed, transdifferentiation (sometimes referred to as adult stem cell plasticity) is a controversial topic in the field of stem cells. It is highly probable that nuclear reprogramming and altered transcriptional activity of important developmental genes are in part responsible for transdifferentiation. Transdifferentiation can be seen as a form of metaplasia due to alterations in the extracellular environment, and often happens during tissue damage [91]. Hence, one probable cause of endometriosis is thought to be due to metaplasia of the peritoneal lining. However, there is also evidence to suggest that circulating stem cells can be a source of metaplastic transdifferentiation when it gains access into the pelvic cavity. Genetically-tagged bone marrow MSCs were tracked in a murine transplant model, and it was established that a small population of it integrated into endometriosis lesions and transdifferentiated into stromal cells (0.1%) and epithelial cells (<0.04%) [92]. It was found that these bone marrow MSCs contributed to the progression of endometriosis rather than initiating it [93]. Changes in cellular phenotype may involve processes that are associated with both embryogenesis and carcinogenesis, such as mesenchymal epithelial transition (MET) and epithelial mesenchymal transition (EMT) [94]. Correspondingly, the invasiveness of endometriotic cells may be attributed to the changes in cellular phenotype in endometriotic lesions. For instance, well differentiated CK^+^E^+^-Cadherin cells, CK^-^E-Cadherin- stromal cells, and an invasive CK^+^E-Cadherin^-^N-Cadherin^+^ epithelial cells (similar to carcinoma micrometastasis) are found in endometriotic lesions [95]. In line with carcinoma characteristics, a regression of endometriotic lesion is seen in estrogen depletion therapy, but recur when therapy stops. This indicates that a quiescent stem cell population is present within the lesion, which reactivates on exposure to estrogen. Thus it may also follow that EnSCs within lesions may also contribute to subsequent lesions.

In terms of regenerative medicine applications, it is envisioned that bone marrow MSCs are more suited for osteogenic differentiation and therefore used for bone tissue engineering, while EnSCs are more suited for soft tissue engineering, such as bladder reconstruction. Due to the intense interest generated with using EnSCs for regenerative medicine, we believe that EnSCs were introduced too quickly into human tests without adequately assessing its impact on large animal models. Furthermore, the exact mechanism of how EnSCs exert is regenerative potential is not clearly understood. Hence more robust large animal studies are needed to fill this information gap that currently exists.

### Concluding remarks and future perspectives

Although EnSCs would form a non-invasive and steady supply of autologous stem cells for women, we must not forget that this means that 50% of the population (i.e. the male population) is excluded. Furthermore, non-invasive harvesting of EnSCs would not be possible from post-menopausal women (in this case, endometrial biopsy might be possible, but data regarding the potency of EnSCs in post-menopausal women has not been published). In addition, the issue of EnSCs storage could also develop into the same debates we are facing regarding the storage of cord blood; is there enough scientific evidence to justify storage of EnSCs for future use?

Nevertheless, given the significant progress made in the application of EnSCs in regenerative medicine, it is envisioned that human clinical trials would be conducted in the near future. Indeed, the RECOVER-ERC trial was recently launched by Medistem Inc (a start-up company based in San Diego, California), which aims to evaluate the potency of EnSCs in 60 heart failure patients in a double-blind placebo controlled Phase II clinical trial [96]. Furthermore, a search in the *clinicaltrials.gov* database reveals that several clinical trials regarding EnSCs are underway, with applications ranging from treating critical limb ischaemia (NCT 01558908), liver cirrhosis (NCT01483248), type I diabetes (NCT01496339), and *in vitro* fertilization (NCT01649752). Hence, it can be envisioned that data from human patients would be available in due course to evaluate the clinical impact of EnSCs.

In conclusion, the discovery of EnSCs represents a paradigm shift for the use of adult stem cells by offering an “off the shelf” therapeutic application regenerative medicine. The EnSCs niche and *in situ* role highlight these cells as being important for cell and tissue regeneration. This work highlights crucial features of an interesting population of mesenchymal progenitors isolated form endometrial tissue. Cells grown *in vitro* were characterized by a high clonogenic potential and a long-term survival. Advantages in comparison with MSC from other sources include a greater ease of supply and the protracted availability during a woman’s lifetime with the additional benefit of deriving stem cells from a waste tissue, thus avoiding critical ethical issues.

In the light of their capacity to differentiate into mesenchymal tissues and their propensity to undergo genetic manipulation, EnSCs hold considerable promise for novel therapeutic approaches for diseases in the wider field of regenerative medicine.

## Competing interests

The authors declare that no competing interests exist.

## Authors’ contributions

JV, AT, AS, AMS conceived and planned the structure of the manuscript. JV, AT, AS, AMS collated and analyzed the data. JV, AT, AS, AMS wrote the manuscript. All authors read and approved the final manuscript.
